# Corrigendum to use of antihypertensive medication and formation of de novo intracranial aneurysms

**DOI:** 10.1111/ene.16381

**Published:** 2024-06-11

Räisänen S, Huttunen J, Huuskonen TJ, von Und Zu Fraunberg M, Koivisto T, Jääskeläinen JE, Frösen J, Lindgren A. Use of antihypertensive medication and formation of de novo intracranial aneurysms. *Eur J Neurol*. 2022 Sep;29(9):2708–2715. doi: 10.1111/ene.15430. PMID: 35652754.

In Table 3, under Model 2, the number of cases for “Regular usage of inhibitors of renin‐angiotensin system” is incorrectly shown as 508/910. It should have been 508/1418.

We apologize for this error.

